# A self-enhanced transport mechanism through long noncoding RNAs for X chromosome inactivation

**DOI:** 10.1038/srep31517

**Published:** 2016-08-16

**Authors:** Chunhe Li, Tian Hong, Chiu-Ho Webb, Heather Karner, Sha Sun, Qing Nie

**Affiliations:** 1 Department of Mathematics, University of California, Irvine, Irvine, CA 92697, USA; 2 Center for Complex Biological Systems, University of California, Irvine, Irvine, CA 92697, USA; 3 Department of Developmental and Cell Biology, University of California, Irvine, Irvine, CA 92697, USA

## Abstract

X-chromosome inactivation (XCI) is the mammalian dosage compensation strategy for balancing sex chromosome content between females and males. While works exist on initiation of symmetric breaking, the underlying allelic choice mechanisms and dynamic regulation responsible for the asymmetric fate determination of XCI remain elusive. Here we combine mathematical modeling and experimental data to examine the mechanism of XCI fate decision by analyzing the signaling regulatory circuit associated with long noncoding RNAs (lncRNAs) involved in XCI. We describe three plausible gene network models that incorporate features of lncRNAs in their localized actions and rapid transcriptional turnovers. In particular, we show experimentally that *Jpx* (a lncRNA) is transcribed biallelically, escapes XCI, and is asymmetrically dispersed between two X’s. Subjecting *Jpx* to our test of model predictions against previous experimental observations, we identify that a self-enhanced transport feedback mechanism is critical to XCI fate decision. In addition, the analysis indicates that an ultrasensitive response of *Jpx* signal on CTCF is important in this mechanism. Overall, our combined modeling and experimental data suggest that the self-enhanced transport regulation based on allele-specific nature of lncRNAs and their temporal dynamics provides a robust and novel mechanism for bi-directional fate decisions in critical developmental processes.

Organisms with the XY mechanism of sex determination have developed dosage compensation mechanisms to balance sex chromosome content between females and males. In mammals, one of the two X chromosomes is transcriptionally silenced in female cells through a process called X chromosome inactivation (XCI). Control of X chromosome activity is crucial for animal development–failure in XCI results in cell death and embryonic lethality^1^. In recent years, key genes have been discovered in regulating XCI, and interestingly, many of these genes encode long noncoding RNAs (lncRNAs), a class of untranslated gene products of which definitive functions were generally elusive. Located in the ‘X-inactivation center’ (*Xic*), a locus on the X chromosome necessary and sufficient for XCI, X-inactive specific transcript (*Xist*) is the master regulator of XCI^2^,^3^. *Xist* upregulation triggers the coating of the inactive X (Xi) by *Xist* RNA and the subsequent silencing of the entire chromosome^4^,^5^. *Xist* expression is controlled by both positive and negative regulators, in particular four lncRNAs located at *Xic*–*Jpx*, *Tsix*, *Xite*, *Tsx*^6^,^7^,^8^,^9^. *Jpx* is transcribed upstream of *Xist* and activates *Xist* by removing CTCF, a transcriptional repressor, from the *Xist* promoter. A molecular titration between the X-encoded *Jpx* RNA and the autosomally encoded CTCF protein defines the X to autosome (X:A) ratio and regulates the initiation of XCI^10^. Together, these lncRNAs represent key elements involved in the allelic regulation at the initiation of XCI.

Despite identification of major regulatory factors, molecular mechanisms for the “counting” of X-chromosomes and the “choice” of Xi remain to be fully elucidated. Dynamic features of lncRNA regulation are underexplored. Several models have been proposed to explain the symmetry breaking processes in XCI: how to choose and keep only one of the two initially active X’s in a female cell to be silenced throughout development. Monkhorst *et al*. used intrinsic stochasticity and feedback mechanism to explain XCI^11^. Another potential mechanism is that the ‘pairing’ between two X chromosomes prior to *Xist* activation is critical for the choice of the X chromosome to be inactivated^12^,^13^,^14^. A two-state switching mechanism of X chromosome was also suggested to explain the random silencing of one X chromosome in XCI^15^.

A class of statistical mechanics model has been proposed based on the “Blocking Factor Hypothesis”, in which a single complex blocks and protects X chromosome from silencing, therefore controlling symmetry breaking in XCI^16^,^17^,^18^. These biophysical models focused on interactions between molecules and the two chromosomes through a “self-assembled” mechanism to induce symmetry breaking. It is difficult to link such models to specific XCI patterns under perturbation of lncRNAs, as well as from perturbation of X chromosome numbers in a cell. For example, XCI is sensitive to the number of X chromosomes relative to the ploidy: a tetraploid cell keeps two active X’s and a triploid cell can have one or two active X’s^19^,^20^. In cases of various X:A ratios (Table 1), the X chromosome number with Xi fate increases as the number of X chromosomes increases, indicating a complicated pattern of XCI depending on X chromosome number. How the X chromosome counting and choice are determined for various X:A ratios remains mostly mysterious^21^.

Here we present a class of models that account for regulatory dynamics of lncRNAs and their signaling properties leading to XCI. Our models are based on two important properties of lncRNAs: fast transcriptional turnovers and spatially localized actions, which make them distinct from proteins or small RNAs^22^. As a result, our model restricts lncRNA molecules only transported between the X chromosomes, and the fast transcriptional turnover allows robust maintenance of the total amount of lncRNA (e.g. *Jpx*) shared by X chromosomes.

Through computational simulations and experimental assays, we tested the three models specifically on the molecular network of lncRNAs *Jpx, Xist,* and the protein CTCF. We found that a self-enhanced transport mechanism on *Jpx* is critical to XCI fate decision for various cases of X:A ratios. The model is supported by the experimental observation on the asymmetric spatial localization of *Jpx* during XCI. In addition, we found that the ultrasensitive interaction between lncRNA (*Jpx*) and protein (CTCF) is essential to a robust decision-making process in XCI.

## Results

At the initiation of random XCI in each female cell, both X chromosomes have the equal chance to be inactivated^23^, and a symmetry breaking mechanism is needed for the asymmetrical fate determination of the two X’s.

### Three models allow the symmetry breaking of XCI for 1X/2A and 2X/2A systems

A molecular mechanism with core regulatory circuit including lncRNAs *Jpx*, *Xist*, and protein CTCF, has been proposed for the initiation of XCI based on previous experimental observations^10^. In this circuit, CTCF protein is an inhibitor of *Xist* RNA, and the binding between *Jpx* and CTCF (forming complex JC) evicts CTCF from the *Xist* promoter, which then activates *Xist*. The upregulation of *Xist* triggers the XCI in the corresponding X-chromosome and eventually silences it. Enlightened by the bi-directional fate decision of *Xist* for the two X chromosomes in each cell^6^,^10^, and the previous reports on how bistablity may induce cell fate switches^24^,^25^,^26^,^27^, we propose three multi-compartment models, each with addition of one specified minimal interaction to the core regulatory circuit that may lead to bistability.

**Figure 1:**
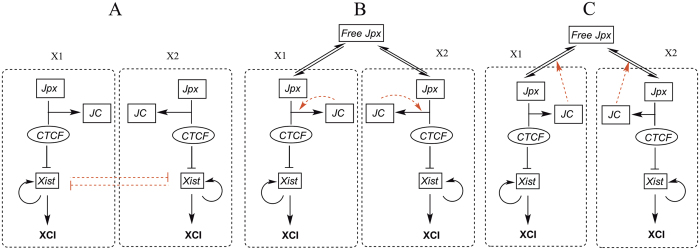
Regulatory circuits for three models on XCI fate decision system. The two dashed rectangular boxes in each model illustrate two separate compartments representing the two X chromosomes. (**A**) Cross inhibition model for XCI. *Xist* at two X chromosomes are self-enhanced and mutually repressed. Two compartments represent two X chromosomes (X1 and X2) separately. JC represents the complex from the binding of *Jpx* and CTCF. (**B**) Self-catalyzed binding model for XCI. The complex JC promotes the binding between *Jpx* and CTCF, which forms a positive feedback loop. (**C**) Self-enhanced transport model for XCI. The complex JC between *Jpx* and CTCF promotes the diffusion for *Jpx*, which forms a positive feedback loop for *Jpx* level in one X chromosome. Black arrows represent activation regulation evidenced from experiments, and black short bars represent repression regulation evidenced from experiments. Red dashed links represent hypothetical regulations proposed in three models.

In the first model, we assume a cross-inhibitory regulation between *Xist* RNA transcribed from the two X chromosomes (red arrows in Fig. 1A) without providing molecular details of such regulation. In the other two models, we assume a constant total amount of *Jpx* transcribed from two X chromosomes but allow *Jpx* RNA to disperse between the two X chromosomes. This is based on the inference that *Jpx* acts both *in cis* and *in trans*, and that lncRNAs can have rapid transcriptional turnover^22^. CTCF is a ubiquitously expressed nuclear protein mostly abundant and stable in mammalian cells^28^,^29^. As previously shown, CTCF-*Jpx* RNA binding removes CTCF from the *Xist* promoter in one X chromosome but does not reduce CTCF occupancy in the opposite X chromosome or in other CTCF-DNA binding sites^10^. We therefore consider the distribution of CTCF as even and independent between the X chromosomes in our models.

In particular, we consider two possible self-enhancement mechanisms derived from the proposed regulation circuit of XCI: a self-catalyzed binding model in which the formation of a *Jpx-*CTCF complex (JC) catalyzes and further promotes the binding between *Jpx* and CTCF (red arrows in Fig. 1B); a self-enhanced transport model in which the binding between *Jpx* and CTCF enhances the diffusion or transport of *Jpx* towards the JC forming locus (red arrows in Fig. 1C). Next, we will simulate each model to study their symmetry breaking abilities for various X:A ratios in cells.

We first examined the cross inhibition (CI) model (Fig. 1A). By running simulations to the steady states, we were able to identify the final XCI fates of different X chromosomes. The CI model replicated the experimental results well for 1X/2A or 2X/2A case (Table 2). The statistical analysis of the stochastic simulations (each simulation represents one cell in fate decision) shows the percentage of the cells that end up in different XCI fates. The accuracy rate, defined as the percentage of cells with the correct XCI fate, is 99% for both 1X/2A and 2X/2A cases.

The second model we tested is the self-catalyzed binding (SCB) model, where a self-catalyzed binding between *Jpx* and CTCF is assumed, i.e. the binding complex formed from *Jpx* and CTCF (JC) will further promote the binding of *Jpx* and CTCF at the same X chromosome (Fig. 1B). We evaluated the performance of SCB model on XCI fate decision patterns at different X:A ratios. For 1X/2A and 2X/2A case (Table 2), the SCB model provided a consistent performance, with an accuracy rate of 99% and 96% separately.

Finally, we studied the self-enhanced transport (SET) model (Fig. 1C) in which the JC binding complex promotes the transport of *Jpx* to its own compartment, for example, through diffusion to the same chromosome, creating a positive feedback loop for *Jpx* dispersion and activity. The simulations of SET model showed the accuracy rate (cell percentage with 1 Xi) for the 2X/2A case at 98%, and the accuracy rate (cell percentage with 0 Xi) for the 1X/2A case at 99%.

The calculated XCI accuracy rates from all the three models were found to be consistent with the previous experiments for the 2X/2A and 1X/2A case (Table 2). However, the mechanisms of symmetry breaking for three models are different. In the CI model, *Jpx* is equally distributed between the two X chromosomes, and the symmetry breaking takes place in the downstream of *Jpx* (cross inhibition between two *Xist*). In the other two models, the symmetry breaking takes place in the upstream of the circuit (interactions between *Jpx* and CTCF). However, the SET model enforces a positive feedback on the *Jpx* transport, not on the binding between *Jpx* and CTCF like SCB model. Such regulation confers SET a better ability for “reallocation” of *Jpx*, which in turn influences fate decision in XCI.

### Fate decision pattern of XCI for multiple X:A ratios supports self-enhanced transport (SET) model

Many of the fate decision patterns of XCI for various X:A ratios have been identified from experiments previously^11^,^21^,^30^,^31^, as shown in Table 1. Evidences collected from these experimental studies of aneuploids and polyploids indicate that tetraploid XXX females (3X/4A) inactivate only one X chromosome and tetraploid XXXX females (4X/4A) inactivate two X chromosomes. In general, XCI in mammals is sensitive to the number of X chromosomes relative to ploidy and follows the rule of one active X chromosome per diploid (2A) set of autosomes^32^. The counting of X chromosomes (positively correlated to the amount of *Jpx* activity) determines the choice of XCI (the number of Xi), and Xi number increases as the number of X chromosomes increases, which indicates a complicated pattern of XCI fate depending on X chromosome number. This suggests that both symmetry breaking and X chromosome dose dependent mechanisms are needed for proper function of XCI fate decision system.

**Figure 2:**
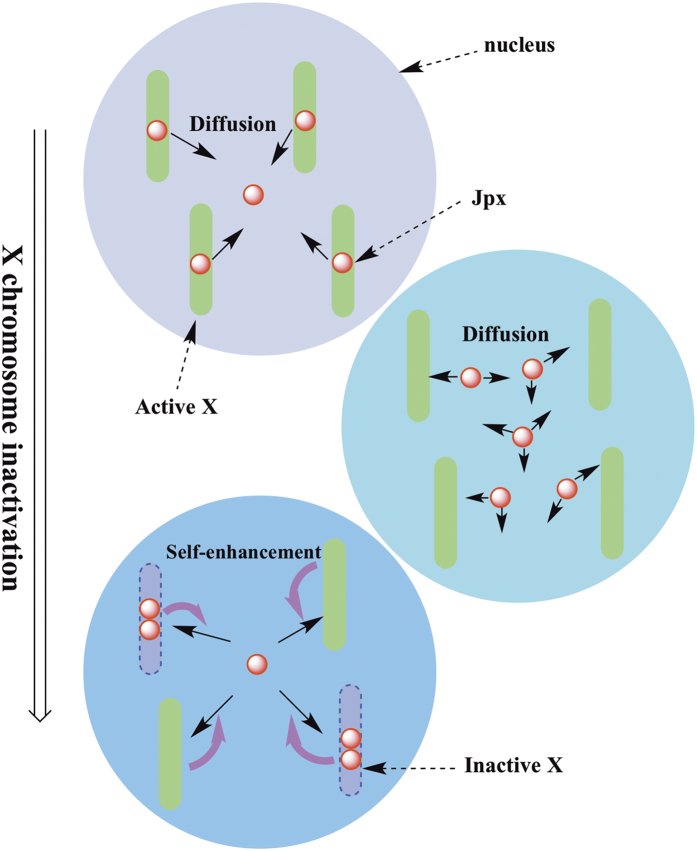
Illustration of the self-enhanced transport mechanism of X chromosome inactivation for multiple X chromosomes, corresponding to the SET model in Fig. 1C. *Jpx*, positively regulating XCI through activating *Xist*, can diffuse among different X chromosomes. In the case of no self-enhancement feedback, all X chromosomes keep to be active because each X has only one *Jpx* (not enough to trigger XCI). The self-enhancement positive feedback makes the symmetry of *Jpx* distribution broken, and causes the “reallocation” of *Jpx* (some X has two *Jpx*, and some X has 0 *Jpx*), leading to disparate XCI fates for different X (two active X and two inactive X in this case). The number of *Jpx* for triggering XCI is not from real data, only for illustration purpose.

To further distinguish among the models, we expanded our models to include more compartments to mimic the multiple X and A cases. In each chromosome of the model, the regulatory circuit is similar to the 2X/2A case. In particular, we compared the accuracy rates (percentage of cells with the correct XCI fate) between simulations and the available experimental data for three models. As demonstrated in Table 2, only the SET model could consistently produce the similar accuracy rates for all X:A ratios that have been measured in experiments. The CI model could not replicate the experimental results for certain X:A ratios, such as the 3X/4A and 4X/4A cases. Comparably, the SCB model failed in the 3X/4A and 2X/4A cases, with accuracy rates of 12% and 4%. With all X:A ratios considered, only the SET model recapitulates the specific XCI patterns observed experimentally. Such results indicate that in order to generate correct XCI patterns, a “global regulation” of the *Jpx* RNA is needed for all X chromosomes, i.e., for each X, *Jpx* may be “recruited” from the other X chromosomes as needed (“*Jpx* reallocation”) (Fig. 2).

Our analysis of the 2X/2A situation showed that all three models could lead to symmetry breaking. However, simulation results for the three models demonstrate that only the SET model leads to the asymmetrical distribution of *Jpx* between the two X chromosomes (Fig. S1). In the CI model, the symmetry breaking in the downstream of *Jpx* (at the *Xist* level) makes the reallocation of *Jpx* impossible. In the SCB model, the positive feedback regulates the binding between *Jpx* and CTCF, not *Jpx* activity, and therefore cannot reallocate *Jpx*. Only the SET model provides the positive feedback that regulates the *Jpx* diffusion directly. As illustrated in Fig. 2, once fluctuations on *Jpx* activity occur among different X chromosomes due to gene expression noise, the positive feedback for *Jpx* diffusion will amplify this asymmetry, and eventually lead to the “reallocation” of *Jpx* activity. The property of *Jpx* RNA re-localizing to the opposite allele has been implicated from previous experimental observations that *Jpx* acts both *in trans* and *in cis*^22^. Such diffusion and allele-specific activities define distinct roles of lncRNAs during development.

To investigate the robustness of the SET model against the fluctuations of parameters, we performed a single variable sensitivity analysis for the parameters under 2X/2A condition (Fig. S2/). By increasing or decreasing each basal parameter by 10%, we calculated the percentage change in the accuracy rate compared with the case using the basal parameter values. We found the percentage changes in accuracy rate upon different parameter perturbations are mostly in the range of −10% to 5%, and only a few of them are close to 20%. We also identified several key parameters that affect the XCI fate decisions more significantly. For example, the synthesis rate of protein CTCF is critical, partly because in our model, CTCF is the major inhibitor for *Xist*, and thus determines the level of *Xist* and the corresponding XCI fate. Another key parameter is the binding rate of *Jpx* and JC1, influencing the activity of final complex JC, which in turn affects the strength of self-enhancement positive feedback, a critical factor for the XCI determination.

### Ultrasensitive response of *Jpx* signal on CTCF is critical to asymmetrical XCI fate decision

**Figure 3:**
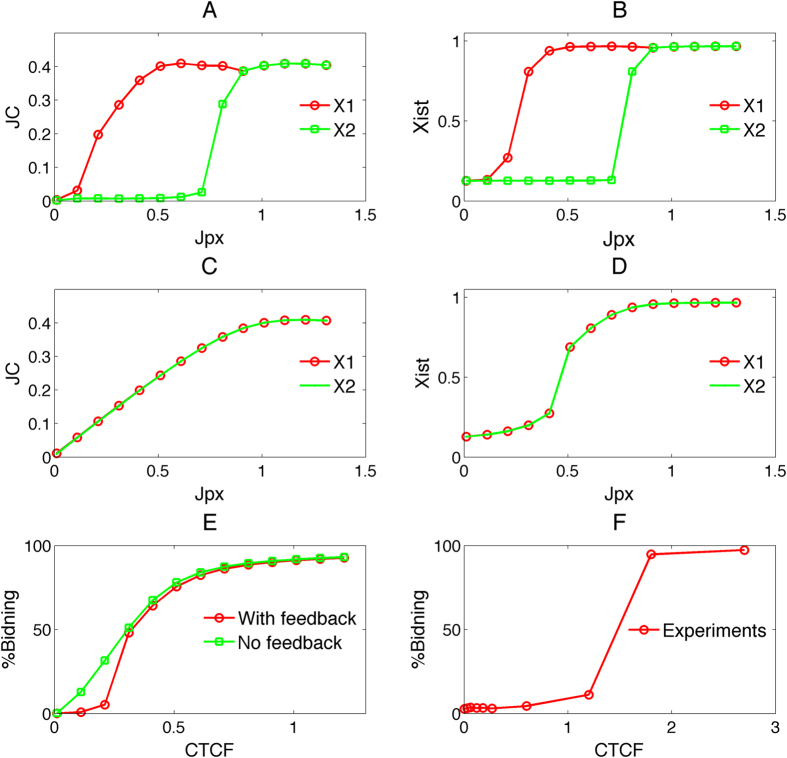
The comparison of signal response curves between the model with self-enhanced transport (SET) and the model without feedback. (**A,B**) Signal response curves between *Jpx* and JC, and between *Jpx* and *Xist* separately for two X chromosomes (X1 and X2) for the model with self-enhanced transport feedback. (**C,D**) Signal response curves between *Jpx* and JC, and between *Jpx* and *Xist* separately for two X chromosomes (X1 and X2) for the model without self-enhanced transport feedback. (**E**) Signal response curves for the binding of CTCF and *Jpx*. Red line: SET model; green line: the model without self-enhanced transport feedback. (**F**) Binding isotherm for CTCF-*Jpx*. Specific binding of *Jpx* lncRNA to CTCF protein was resolved by RNA gel electrophoresis mobility shift assay (EMSA) (Fig. S3). The binding curve was plotted as the percent bound against CTCF concentration.

One major property in the SET model is the self-enhanced regulation of the JC complex on the *Jpx* transport, thus we next examined the function of this regulation in terms of the activation kinetics. By running simulations with various concentrations of *Jpx*, we first obtained the relationship between the amount of *Jpx* (signal) and the amount of JC complex formed (response), and we compared the signal-response curves in the model with self-enhanced transport (SET model) and a control model without this regulation (Fig. 3A,C). We found that the SET feedback model leads to sigmoidal binding curves (Fig. 3A), which indicates an ultrasensitive or abrupt accumulation of JC in response to increasing *Jpx* concentrations, i.e. a switch-like behavior, whereas the control model only leads to linear-like binding curves (Fig. 3C). Similarly, we examined the signal-response relationship between *Jpx* concentration and *Xist* concentration, and we found that both models generate sigmoidal signal-response curves (Fig. 3B,D). However, for the model without the SET feedback two response curves for two X chromosomes are indistinguishable (Fig. 3D), whereas the SET feedback model leads to two distinct curves that represent the contrasting activation kinetics of *Xist* in the two X chromosomes (Fig. 3B). In particular, the two X chromosomes have distinct activation thresholds in response to *Jpx* (Fig. 3B). This provides a further support that self-enhanced transport is critical for the symmetry breaking mechanism in asymmetrical XCI fate decisions.

We also examined CTCF-*Jpx* binding kinetics by evaluating the signal-response relationship between CTCF concentration and the percentage of bound CTCF in the SET model (with the SET feedback) and the control model (no SET feedback) (Fig. 3E). The signal-response curve in the SET model shows a sigmoidal binding, whereas the control model only shows a hyperbolic curve (Fig. 3E). To experimentally resolve the binding activity of *Jpx* and CTCF, we performed electrophoresis mobility shift assay (EMSA) using *in vitro* transcribed *Jpx* RNA and purified recombinant CTCF protein (Fig. S3). Binding isotherm for *Jpx*-CTCF was determined by titrating the concentration of protein added to radiolabeled RNA and calculating the fraction of bound complex JC. Our experiments indicate that the binding between CTCF and *Jpx* indeed exhibits a sigmoidal signal-response curve (Fig. 3F), indicating highly cooperative interactions. Together, our results from simulations and experiments suggest that an ultrasensitive response between *Jpx* and CTCF is critical to the bi-directional fate decision of XCI.

### *Jpx* is transcribed biallelically, escapes XCI, and is asymmetrically dispersed between two X’s

**Figure 4:**
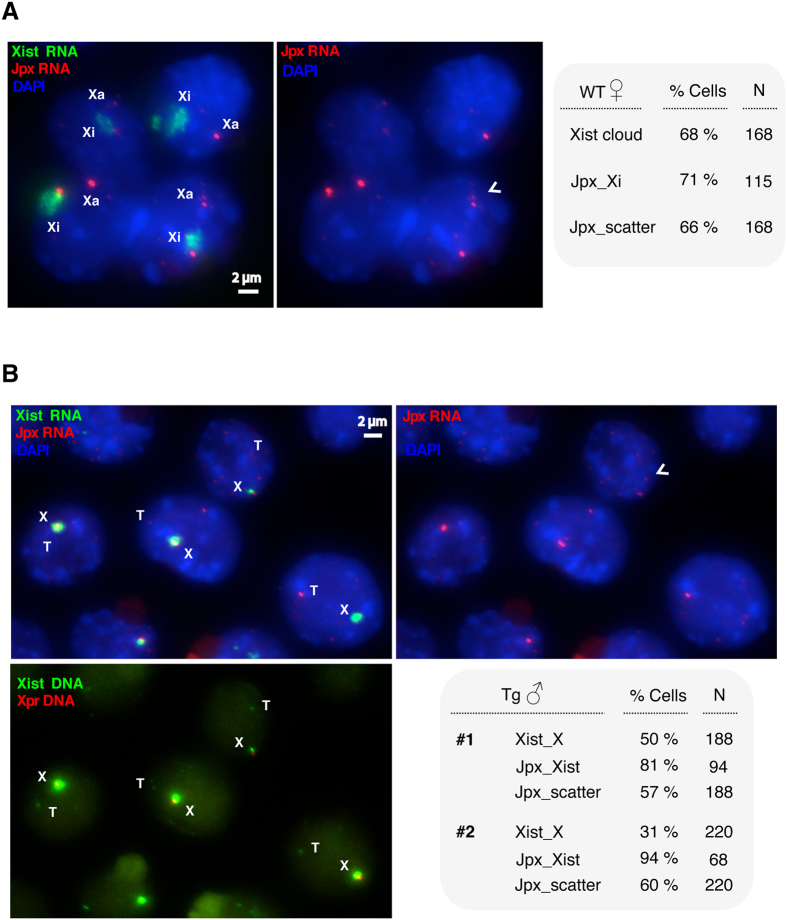
Biallelic expression and dispersed *Jpx* RNA in differentiating ES cells. (**A**) RNA FISH detecting *Jpx* and *Xist* expression in wild-type female cells during ES cell differentiation at Day 8. *Xist* cloud (green) is associated with Xi (the inactive X chromosome). *Jpx* RNA (red) is present on both Xi and Xa (the active X chromosome). Quantitation of cells is shown for the presence of *Xist* cloud, the *Jpx* RNA on Xi, and the scattered *Jpx* RNA in the cell nucleus. (**B**) Endogenous *Xist* is upregulated in male ES cells carrying a *Jpx-Xist* transgene, as examined by RNA FISH in the transgenic male cells during ES cell differentiation at Day 2. DNA FISH was performed for *Xpr* (X-pairing region, red, bottom panel), which marks the endogenous *Xic* (X-inactivation center) and therefore the X chromosome^48^. *Xist* upregulation (green) is associated with the endogenous allele on X. *Jpx* RNA (red) is present with the active *Xist*. T, transgene; X, X chromosome. Quantitation of cells from two transgenic male ES clones (#1 and #2) is shown for the presence of *Xist* upregulation on X, the *Jpx* RNA associated with *Xist,* and the scattered *Jpx* RNA in the nucleus. Arrowheads mark dispersed *Jpx* RNA. N, sample size.

To test if *Jpx* molecules are unevenly distributed between the two X chromosomes, we performed corresponding experiments. The molecular programing of XCI can be closely examined during the time-course analysis of mouse embryonic stem (ES) cell differentiation. As a “numerator” for X chromosome counting, *Jpx* escapes XCI and is transcribed from both X chromosomes during female ES cell differentiation^6^. We analyzed the expression patterns of *Jpx* and *Xist* in wild-type female ES cells using RNA fluorescent *in situ* hybridization (FISH). As shown in Fig. 4A, *Xist* upregulation formed characteristic “cloud” in the cell nucleus at ES differentiation Day 8 and specified the inactive X chromosome (Xi). In such cells, 71% showed the *Jpx* RNA on Xi, consistent with the previous analysis^6^. In addition to the biallelic transcription of *Jpx*, we observed that *Jpx* transcripts were scattered and seemed to be unequally distributed between the two alleles.

To determine if asymmetric allocation of the diffusible *Jpx* RNA regulates *Xist* activation, we examined male ES cells carrying an autosomal *Jpx-Xist* transgene. Because *Xist* is normally not upregulated in male cells, activation of the endogenous *Xist* RNA must be caused by the transgene. We characterized the expression of *Jpx* and *Xist* at ES differentiation Day 2, an early time point for XCI initiation. As shown in Fig. 4B, *Xist* upregulation was observed as robust RNA “domain” in the cell nucleus. Serial RNA-DNA FISH was performed on the same cells to determine the allelic expression. Consistent with a *trans*-regulatory effect, upregulation of the endogenous *Xist* was observed in 31–55% of the transgenic male cells. In such cells, 81–94% showed the *Jpx* RNA associated with *Xist* transcription. At the same time, 57–60% of the cells exhibited *Jpx* transcripts scattered and unevenly distributed between the alleles. Therefore, our experimental observations support the asymmetrical distribution of *Jpx*, and the SET mechanism for XCI.

### A transient and switch-like activation in XCI

**Figure 5:**
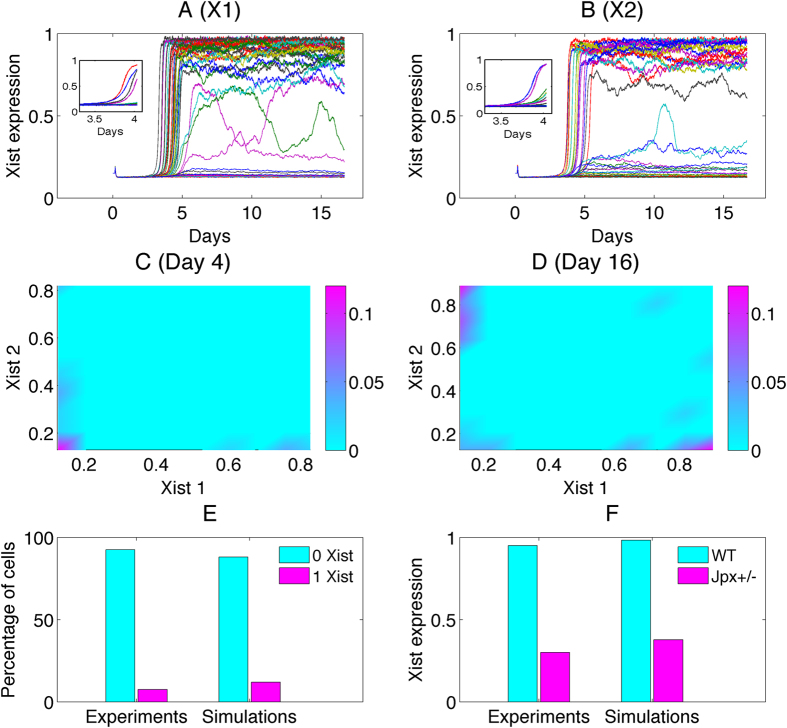
Trajectories and distributions of *Xist* RNA for Day 4 and Day 16. (**A,B**) Trajectories for *Xist* RNA expression level at two X chromosomes from day 0 to day 16 obtained from SET model. Fifty cells are plotted, and each trajectory represents a cell in fate decision. The inset shows the trajectories for day 4. X1 represents X chromosome 1, and X2 represents X chromosome 2. (**C,D**) Distributions of cells for Xist 1 (*Xist* level at X chromosome 1) and Xist 2 (*Xist* level at X chromosome 2) at day 4 and day 16. At day 4, the cell population is mostly distributed on the left bottom corner, indicating that most of cells express neither Xist 1 nor Xist 2. For day 16, the cell population is mostly distributed in the right bottom or left top corner, indicating that for most of cells one out of the two X chromosome expresses *Xist*. (**E**) The comparison between experiments and simulations from SET model for the percentage of cells with 0 *Xist* cloud and 1 *Xist* cloud at day 4. (**F**) The comparison between experiments and simulations for the relative *Xist* RNA level for *Jpx* knockout at Day 12. Cyan bars represent wild- type, and magenta bars represent one *Jpx* is knocked out.

Next we performed simulations for *Xist* expression level from day 0 to day 16 of mouse ES cell differentiation. Each trajectory represents the stochastic expression dynamics for one cell. On day 16 (Fig. 5A,B), the system is close to the steady state, and *Xist* expressions display two different levels, which further lead to two different XCI fates. However, on day 4 (the inset of Fig. 5A,B), the *Xist* expression levels show large differences from those on day 16. This is because day 4 represents the early stage of XCI—initiation of *Xist* upregulation occurs but only in a small percentage of cells. The percentage of cells with *Xist* expression in one X is very low (about 11% as shown in Fig. 5E). Additionally, *Xist* temporal trajectories show that for most of cells the *Xist* activation happens between day 4 and day 7 (Fig. 5A,B). This implicates a transient and switch-like activation in XCI. The switch happens spontaneously because of the bistable properties of the dynamical system in the model.

Previous experiments showed that deleting a single allele of *Jpx* abolishes XCI^6^. We performed simulations with SET model to mimic knocking down a *Jpx* allele, by decreasing the total *Jpx* amount to the half as in wild-type. We compared the relative *Xist* RNA levels from the experimental data and simulations (for Day 12), as shown in Fig. 5F. The simulations, which agree with the experimental data^6^, show that the *Jpx* knockout will reduce *Xist* expression significantly (Fig. 5F). Additionally, we found from experiments that *Xist* is highly expressed on Day 12 for the wild-type (cyan bar in Fig. 5F), in opposition to only slight expression on Day 4 (magenta bar in Fig. 5E). This underpins the transient and switch-like activation in XCI suggested in our model, and implies that the switch behavior happens between day 4 and day 12, consistent with our model predictions.

Figure 5C,D show the probability distribution of cell populations with the coordinate of Xist 1 (*Xist* at X chromosome 1) and Xist 2 (*Xist* at X chromosome 2) separately for day 4 and day 16 obtained from SET model. At day 5, most of cells express neither Xist 1 nor Xist 2 (Fig. 5C), i.e., the percentage of cells with *Xist* expression in both X’s is low. At day 16, one of the two X chromosomes expresses *Xist* in most of cells (Fig. 5D). Experimental data on the XCI fate decision at day 4 are in good agreement with our simulation results^6^ (Fig. 5E).

**Figure 6:**
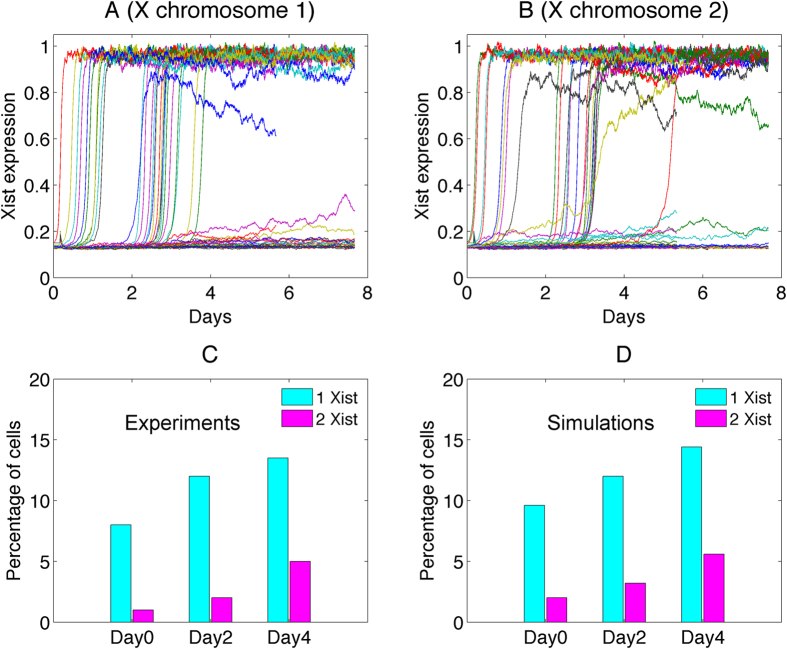
The comparison between simulations and experiments for *Jpx* transgenic female ES cells. (**A,B**) Trajectories for *Xist* RNA expression level at two X chromosomes from day 0 to day 8 obtained from SET model for adding transgene (*Jpx*). (**C,D**) The comparison between experiments and simulations for the percentage of cells with different number of *Xist* clouds for adding transgene (*Jpx*).

Previous experiments also showed that *Jpx* overexpression results in ectopic *Xist* upregulation^10^. We compared the experimental observation and computer simulations for the *Jpx* transgenic female ES cells (Fig. 6). In most cells, *Xist* is upregulated between day 0 to day 4 under this transgenic condition, suggesting an accelerated XCI activation compared to the wild-type (Fig. 6A,B). On the population average, the percentage of cells with *Xist* expression in one X and *Xist* expression in two X’s gradually increase with time (from day 0 to day 4) as seen in Fig. 6C,D, which also shows the agreement between simulations and experiments.

A recent study challenged the trans-acting role of *Jpx* in XCI. Large X-chromosome deletions including *Jpx* and other elements affected XCI only mildly, and a transgene carrying *Jpx* failed to rescue the X-inactivation defects^33^. Differences of *Jpx* RNA levels in these deletion and transgenic cells may be directly relevant to the activity of *Jpx* on *Xist*. However, the study proposed a different mechanism with the trans-acting activator of *Xist* being the X-encoded E3 ligase protein RNF12, which cooperates with lncRNAs *Jpx, Ftx,* and *Tsix,* to induce *Xist* upregulation^33^,^34^.

### RNF12 accelerates the dynamics of XCI

**Figure 7:**
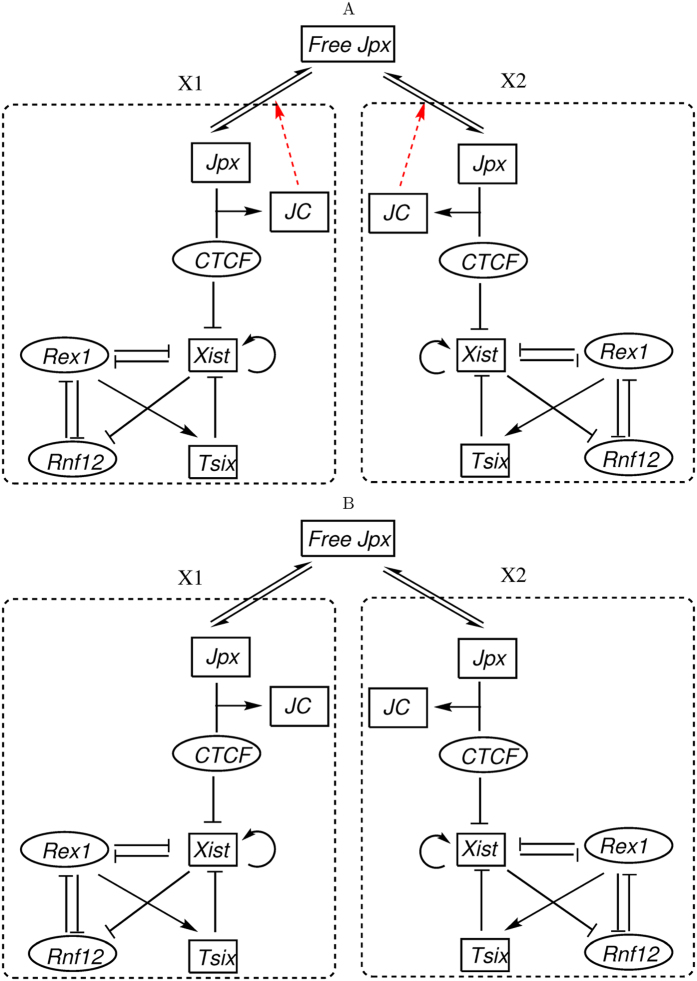
Regulatory circuits for the SET model adding Rnf12, Rex1, and Tsix related regulations. Two compartments represent two X chromosomes (X1 and X2) separately. JC represents the complex from the binding of Jpx and CTCF. (**A**) The expanded SET model (ESET) with the self-enhanced diffusion for Jpx, assuming that the complex JC between Jpx and CTCF promotes the diffusion for Jpx, which forms a positive feedback loop for Jpx level in one X chromosome. (**B**) The model without self-enhanced diffusion for Jpx (WSET). Black arrows represent activation regulation evidenced from experiments, and black short bars represent repression regulation evidenced from experiments. Red links represent hypothetical regulations proposed in the model. Rectangle nodes represent long noncoding RNA, and ellipse nodes represent protein.

Indeed, *Tsix* and RNF12 have been suggested as critical factors in XCI fate decisions^6^,^33^,^35^,^36^. We incorporated the regulatory activity of *Tsix* and RNF12 in our SET model and constructed an expanded (more comprehensive) SET circuit (ESET, Fig. 7A).

Simulations based on the ESET model demonstrate that the addition of RNF12 and *Tsix* does not affect the XCI fate decisions dramatically. As shown in Table 3, 96% cells from simulations resulted in the correct XCI fate decision for 2X/2A (one of the two X chromosomes is inactivated), as long as the model possesses the self-enhanced transport regulation for *Jpx*. However, if the self-enhanced transport regulation is removed from the model (Fig. 7B), simulations show that only 28% cells led to the correct XCI fate (Table 3). As such, these results underscore the essential role of the self-enhanced transport regulation for XCI fate decision.

To determine the role of RNF12 on XCI fate decision with this model, we removed the RNF12 node from the ESET circuit (Fig. 7A), mimicking an RNF12 knockout, and examine how the *Xist* dynamics is influenced. Comparison between two individual typical trajectories from the simulation shows that the steady state expression level of *Xist* decreases, and the activation time for *Xist* becomes longer after the RNF12 knockout (Fig. S4). This is because that RNF12 activates *Xist* through REX1 (RNF12 inhibits REX1, and REX1 inhibits *Xist*)^34^, and thus the knockout of RNF12 will reduce the expression level of *Xist*. On the other hand, *Xist*, RNF12, and REX1 form a negative feedback loop (RNF12 inhibits REX1, REX1 inhibits *Xist*, and *Xist* silences RNF12). The negative feedback loops have been suggested to accelerate the response time^37^, which provides an explanation for RNF12 knockout slowing the activation time of *Xist*. Overall, these results show that RNF12 influences the dynamics of *Xist* activation through a negative feedback loop, which likely provides a way to regulate the activation timing of XCI. This is consistent with the *in vivo* analysis of RNF12 knockout mice exhibiting no apparent defects in random XCI^38^ and the study of RNF12 heterozygous deletion in female ES cells showing delayed XCI^36^.

## Discussion

X Chromosome Inactivation (XCI) has been studied extensively using various approaches^39^,^40^. However, the underlying molecular mechanisms for the counting and choice of XCI at various X:A ratios remain to be elucidated. By incorporating the distinct features of lncRNAs in their localized actions and fast turnovers, we built three models and found all of which can explain the XCI fate decision patterns for the 2X/2A and 1X/2A cases. In particular, we found that only the self-enhanced transport (SET) model, incorporating a positive feedback on the lncRNA *Jpx* regulated signal, can best replicate XCI patterns observed in experiments for the multiple X and A cases. The *Jpx* self-enhanced transport mechanism helps to determine the formation of correct XCI patterns, especially for multiple X’s. The transport of *Jpx* highlights the local property of lncRNA (*Jpx* acts *in cis* and *in trans*), and the positive feedback loop resulted from the self-enhanced transport mechanism is closely connected with the ultrasensitive binding between lncRNA *Jpx* and protein CTCF. Together, they represent distinct regulatory features and imply unique roles of lncRNAs in XCI. To our knowledge, this is the first mathematical model embracing the molecular features of lncRNAs in XCI fate decision, which recapitulates XCI patterns observed from experiments for all X:A ratios.

The asymmetric distribution of *Jpx* between the two X chromosomes observed in our experiments further supports the SET model as the key mechanism in inducing different *Jpx* activity at two X chromosomes, or disparate expression of *Xist*, leading to divergent XCI fates. Because the two homologous X chromosomes are essentially identical with the same set of regulatory elements, and RNA molecules can diffuse between the X chromosomes rapidly, *Jpx* activities are considered similar between the two X’s if there is no “reinforcement” mechanism. In the SET mechanism, once *Jpx* RNA reach a threshold level in one X chromosome due to fluctuation, more *Jpx* bind to CTCF and form JC complex because of a positive feedback, which further enhance the flow of *Jpx* into the same X chromosome. As a result, *Xist* is activated and the corresponding X chromosome is inactivated. In contrast, *Jpx* level in the other X is reduced due to rapid transcriptional turnover, which renders it insufficient to trigger *Xist* activation and XCI. When more X chromosomes are present in the cell, a *Jpx* “reallocation” mechanism is needed to reach the correct XCI patterns according to various X:A ratios, i.e., *Jpx* molecules are recruited from opposite X’s for the activation of *Xist* and XCI (Fig. 2). The positive feedback confers the symmetry breaking ability to this system, and the diffusion property of *Jpx* makes the recruiting process of *Jpx* possible. The combined mechanism thus ensures a robust *Jpx* “reallocation” and the formation of correct XCI patterns.

Intuitively, the self-enhanced transport mechanism creates a stochastic bistable switch in each X chromosome. A higher level of *Jpx* could appear in either of the two X chromosomes, leading to opposite XCI fates for the two X’s. This is consistent with the XCI pattern of most female cells in placental mammals including humans, for which the choice of which X chromosome will be inactivated is random. For the multiple X and A cases, the self-enhanced transport mechanism has some similarity to the classical self-organization (Turing) patterning mechanism in which a short range activation (i.e. positive feedback via activated transport) is coupled with a long range substrate depletion (*Jpx* depletion near the active X chromosome)^41^. While the SET model might not be able to generate stable periodic patterns like the Turing mechanism, it is worth noting that such similar strategy for spontaneous symmetry breaking is shared by biological systems for two totally different purposes: organizing different types of cells in space for patterning and controlling different gene expression patterns in different chromosomes inside one cell.

From our simulation, the SET model suggests a sigmoidal signal response curve (ultrasensitivity) for the binding between *Jpx* and CTCF, which is supported by experiments. Interestingly, our experiments and simulations show that the XCI activation (*Xist* level) also exhibits an ultrasensitive (switch-like) response. The ultrasensitivity in *Xist*, induced by the ultrasensitivity in the binding between *Jpx* and CTCF, can produce abrupt response, which is critical to the symmetry breaking and the formation of XCI pattern for multiple X’s.

We would like to emphasize that a major point in our work is to provide a possible mechanism (self-enhanced transport, or SET) that can explain the symmetry breaking of XCI and can replicate the experimental observations of XCI patterns for various X:A ratios. The key element in the SET mechanism is the self-enhanced positive feedback. Importantly, we find that *Jpx* is the best candidate, among all known regulators so far, for the SET mechanism applicable to XCI. Specifically, the assumptions of an overall constant for the initial amount and the diffusibility of the molecules are critical for the SET model to successfully replicate the experimental observations of symmetry breaking in XCI. Between *Jpx* and *Tsix*, two lncRNA regulators of *Xist*, *Jpx* has been shown to act both *in cis* and *in trans*^6^,^22^. In this study, we have demonstrated that *Jpx* indeed is transcribed biallelically and is diffusible between alleles (Fig. 4), which supports *Jpx* as the primary candidate for SET. In contrast, *Tsix* is known for its direct suppression of *Xist* through antisense transcription entirely *in cis*^42^.

Our expanded SET model absorbing RNF12 and Tsix regulations shows that the RNF12-induced negative feedback loop actually accelerates the activation of *Xist*, which may provide a mechanism for regulating the activation timing of XCI. Indeed, while XCI is associated with ES cell differentiation, the progression of XCI is strongly linked with embryonic development and pluripotency^43^,^44^. Many stem cell factors (such as OCT4 and Nanog) have been suggested to repress *Xist* expression^45^. During early embryonic development, the upregulation of *Xist* is accompanied by the downregulation of these stem cell factors. As more molecular details on XCI become available, it is necessary to develop a more comprehensive XCI model that takes into account the interplay between XCI and stem cell differentiation, in particular, on the roles of lncRNAs in such interplay. Such integrated experimental and modeling study will have great potential of improving our understanding of the regulatory principles and mechanisms for stem cell differentiation and XCI.

We believe that the self-enhanced transport mechanisms we proposed here are general, and should not be dependent on specific molecular details. Therefore, the similar mechanisms as we suggested here are applicable to XCI related and other biological processes involving symmetry breaking or alternative fate decisions.

## Methods

### Simulation methods

Based on the regulatory circuits of three models, we construct the ordinary differentiation equations (ODEs) separately for the three models based on Hill cooperativity form representing activation or repression regulations^46^. Essentially, the ODE models include three terms, which separately denote basal synthesis rates, activation or repression from other genes or proteins, and self-degradations. Next, by adding a noise term (white noise) to the ODE model, we obtain a stochastic ordinary differentiation equation (SDE) for the effects of fluctuations on the regulatory dynamics of the XCI fate decision system.

By solving the ODE/SDE numerically, we are able to identify the final XCI fates of different X chromosomes. We carry out multiple simulations with different random initial conditions to mimic the different XCI fate of multiple cells. For example, for the 2X/2A case, we run 1000 times of simulations to mimic the XCI fate decision process of 1000 cells, and obtain the statistical results for their different XCI fates. The accuracy rate of XCI fate decision is defined as the percentage of cells with the final correct XCI fate based on Table 1. The SDE is solved until time *t*_*f*_  =  1000 using the Euler-Maruyama method with step-size *dt* = 0.01^47^.

## Experimental Methods

### Cell culture and ES differentiation

Culture conditions and ES differentiation methods for wild type 16.7 female (40XX) and *Jpx* knock out ES cells are described in previous work^7^. Briefly, ES cells were grown in culture media plus 500 U/mL of LIF (leukemia inhibitory factor) on a layer of mouse embryonic fibroblast feeder cells to maintain stemness. Upon differentiation, ES cells were separated from feeder cells and LIF was removed from culture media. Cells were collected on day 0, 4, 8, and 12 of differentiation.

### RNA FISH

RNA FISH method has been described previously^7^. For *Xist* RNA-FISH, we used a fluorescein-12-dUTP-lableled Sx9 probe. For *Jpx* RNA-FISH, we used a Cy3-labled DNA probe made from a plasmid containing the full-length *Jpx* gene. For serial RNA-DNA FISH, RNA FISH was performed first. Slides were postfixed in 4% (wt/vol) paraformaldehyde, treated with RNase A, and denatured. For *Xpr* DNA-FISH, we used a Cy3-labled DNA probe made from BAC5 DNA outside the *Jpx*/*Xist* sequence^48^.

### RNA EMSA

EMSA was carried out using *in vitro* transcribed body labeled RNAs and purified CTCF protein. DNA template for *Jpx in vitro* transcription was amplified from mJpx E1-E3 plasmid^10^ using primers JW21F: TTCCCGCGAAATTAATACGACTCACTATAgggagCCACGGCACCACCAGGCTTC and JW22R: GAGTTTATTTGGGCTTACAG. Briefly, radiolabeled RNAs were incubated with titrated concentrations of protein, resolved in polyacrylamide gel electrophoresis (PAGE), exposed to phosphorimage screen (GE Healthcare), and analyzed using Typhoon phosphorimager and ImageQuant software (GE Healthcare). The fraction of bound complex JC was plotted against the concentration of CTCF.

### Transgenic ES cell lines

A 120kb transgene containing the full-length *Jpx* in its endogenous genomic context with *Xist* was made by ET-cloning from BAC 388K20. Transgenic ES cells were generated by electroporation with stable clones picked after 8–11 days under antibiotic selection. Transgene integration was confirmed by DNA FISH and activation of *Xist* was analyzed by RNA FISH.

## Additional Information

**How to cite this article**: Li, C. *et al*. A self-enhanced transport mechanism through long noncoding RNAs for X chromosome inactivation. *Sci. Rep.* **6**, 31517; doi: 10.1038/srep31517 (2016).

## Supplementary Material

Supplementary Information

## Figures and Tables

**Table 1:** XCI fate decision pattern for different X:A ratios indicated from experiments^21^.

X/A	X chromosome Number with Xi	Experiments
1X/2A	0	
2X/2A	1	92%^11^
3X/2A	2	
4X/2A	3	30% or 70%^30^
3X/3A	1 or 2	
2X/4A	0	90%^31^
3X/4A	1	
4X/4A	2	

**Table 2:** Comparisons of three models for the XCI fate decisions for different X:A ratios.

X/A	Xi number from experiments	CI	SCB	SET
1X/2A	0	99%	99%	99%
2X/2A	1	99%	96%	98%
3X/2A	2	64%	52%	82%
4X/2A	3	74%	10%	74%
3X/3A	1 or 2	2% or 76%	24% or 50%	64% or 36%
2X/4A	0	99%	12%	99%
3X/4A	1	6%	4%	99%
4X/4A	2	28%	56%	90%

CI represents cross inhibition model, SCB represents self-catalyzed binding model, and SET represents self-enhanced transport model.

**Table 3:** Comparisons of three models for the XCI fate decisions at 2X/2A case.

Xi number for 2X/2A	SET	ESET	WSET
0	1%	0%	40%
1	98%	96%	28%
2	1%	4%	32%

SET represents self-enhanced transport model, ESET represents the expanded SET model absorbing RNF12 related regulations (Fig. 7A), and WSET represents the expanded model without self-enhanced transport regulation of *Jpx* (Fig. 7B).
