# Supplementary material for: A self-enhanced transport mechanism through long noncoding RNAs for X chromosome inactivation
Source: Sci Rep. 2016 Aug 16;6:31517. doi: 10.1038/srep31517 (PMC4985753; doi:10.1038/srep31517)
Supplement: Supplementary Information [file srep31517-s1.pdf]

Supporting Information for: A self-enhanced transport mechanism through long noncoding RNAs for X chromosome inactivation

Chunhe Li[1,2], Tian Hong[1,2], Chiu-Ho Webb[3], Heather Karner[3], Sha Sun[2,3,*], and Qing Nie[1,2,3,*]
1 Department of Mathematics, University of California, Irvine, Irvine, CA 92697, USA
2 Center for Complex Biological Systems, University of California, Irvine, Irvine, CA 92697, USA
3 Department of Developmental and Cell Biology, University of California, Irvine, Irvine, CA 92697, USA
∗ E-mail: shasun@uci.edu, qnie@math.uci.edu

Parameter setting for ordinary differential equations

In this work, we set the parameter values in the model based on the following:

1, We chose parameter values according to some previous works on gene regulatory networks [1–3]. For example, the Hill coefficient usually reflects the degree of the cooperativity. In this work we chose Hill coefficient n = 4 to represent high cooperativity of gene regulations.

2, To reduce the complexity of the model, we set most of the parameters uniformly (i.e. the different proteins or RNA have the similar synthesis or degradation rate values unless we have direct experimental evidences showing otherwise) because it lacks quantitative experimental data for those values in the XCI network. For example, we set all the degradation rate as k = 1, most basal synthesis rate as a = 1, the Hill coefficient as n = 4. Of note, we used non-dimensional parameter values in our models, because so far there are no sufficient experiment data guiding us to determine the precise value of these parameters.

3, We set parameter values that can satisfy certain biological constrains, including generating steady state solutions as well as generating bistability. This is because XCI corresponds to a bistable switch (Xist ON and Xist OFF).

4, We performed the sensitivity analysis to all the parameters, shown in the Results section (Figure S2), which supports the robustness of current parameter regions for our models.

The illustration for the binding between Jpx and CTCF and the initial values of the models

For the binding dynamics of Jpx and CTCF, we assume that one Jpx molecule binds to two CTCF molecules, i.e. Jpx binds to CTCF forming the the complex JC1, and JC1 binds to CTCF forming the complex JC2 (JC in the main text). So, the reactions for the binding between Jpx and CTCF take the following forms: $Jpx + CTCF \underset{f1}{\overset{h1}{\Longleftrightarrow}} JC1$, $JC1 + CTCF \underset{f2}{\overset{h2}{\Longleftrightarrow}} JC2$. Here the double arrows represent the reversible binding reactions.

Additionally, we chose the initial values of different proteins or RNAs as the following: $I_{Xist} = 0.1, I_{Jpx} = 0.005, I_{ctcf} = 0.1, I_{CTCF} = 0.1, I_{JC1} = 0.001, I_{JC2} = 0.001$. Here I_x (x refers to different RNAs or proteins) represent the initial values. To consider molecular fluctuations within the cell, we gave the initial values a perturbation level ($pl = 0.001$, i.e. the initial value is taken from a probability distribution with a mean of I_x, and a standard deviation given by $\sigma = pl * I_x$) to repeat the simulations multiple times (each simulation represents the XCI process for one cell). In this way, each simulation actually starts with different initial values, and the average of multiple simulations produces the XCI outcome for the entire cell population (the percentage in Table 2 of matin text).

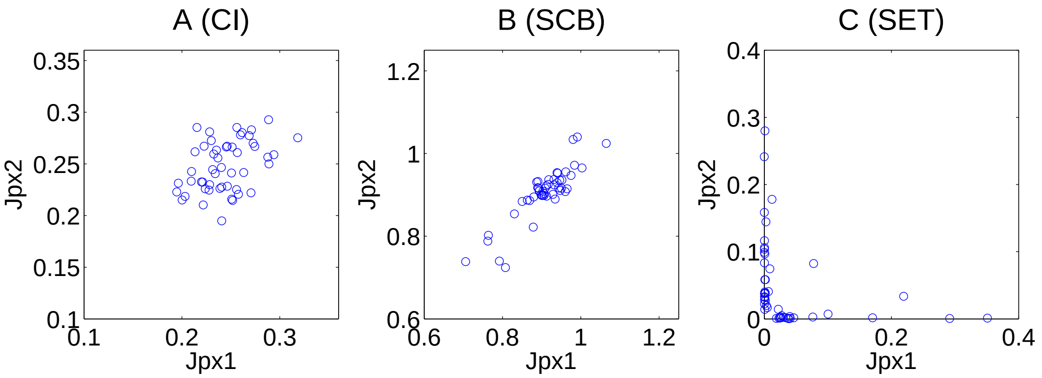

Figure S1. The *Jpx* distribution of cell population at two X chromosomes for three models. Jpx1 and Jpx2 separately denote the relative *Jpx* expression level for two X chromosomes. Each circle represents a cell in steady states. CI represent cross inhibition model, SCB represents self-catalyzed binding model, and SET represents self-enhanced transport model. For CI (A) and SCB (B) models, cells are distributed around the diagonal, indicating symmetrical distribution of *Jpx* between two X chromosomes. For SET model (C), cells are distributed around the X and Y axis, indicating asymmetrical distribution of *Jpx* level between two X chromosomes.

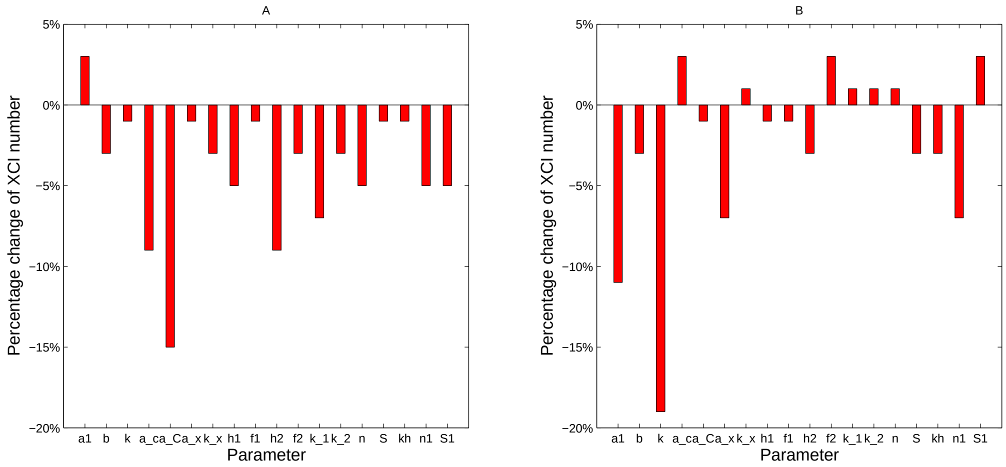

Figure S2. Sensitivity analysis of parameters for SET model. (A) Parameters are increased 10%. (B) Parameters are decreased 10%. By increasing or decreasing each parameter 10%, we calculated the percentage change for the accuracy rate (percentage of cells with XCI happening in one X chromosome) from the model with the default parameter values. Our sensitivity analysis shows that the model is stable against parameter fluctuations at current parameter region. The percentage changes in accuracy rate for different parameter perturbations are mostly in the range of (-10% to 5%), and a few of them are close -20%. We also found some key parameters that affect the XCI fate for different chromosomes critically. For example, a prominent parameter is a_C (synthesis rate of protein CTCF). This is reasonable because that CTCF is the major inhibitor for Xist, which critically determines the level of Xist and the corresponding XCI fate. Another key parameter is h2, which represents the binding rate of Jpx and JC1 (complex from Jpx and CTCF). The binding rate h2 influences the activity of final complex JC2, which affects the strength of self-enhanced positive feedback, and therefore influences the behavior of the XCI system crucially.

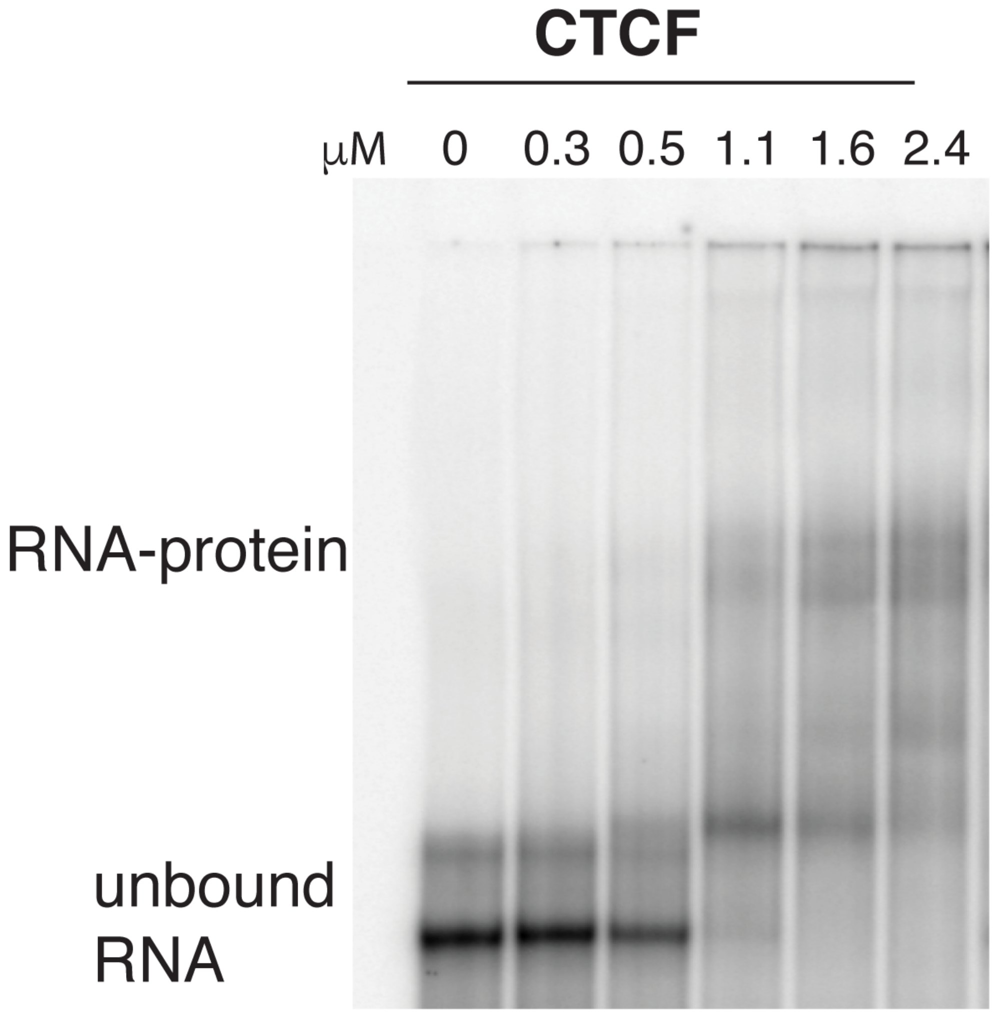

Figure S3. RNA electrophoresis mobility shift assay (EMSA) for direct binding of Jpx RNA and CTCF protein. RNA preparation and EMSA were performed as previously reported [4, 5] with additional details described in the Methods. Titration of a 383nt Jpx RNA with CTCF protein was conducted with an increasing concentration of protein. Samples were resolved on a 5% native acrylamide gel. The gel was dried and placed in a phosphor-imaging cassette for exposure. The image was scanned with a Typhoon phosphor imager and analyzed with the Image QuantTL software. The signal intensities were quantified, and the fraction of bound RNA was obtained for each CTCF concentration. The RNA-protein binding kinetics can be obtained by performing a nonlinear regression in Microsoft Office Excel using percent bound RNA against the protein concentration [6].

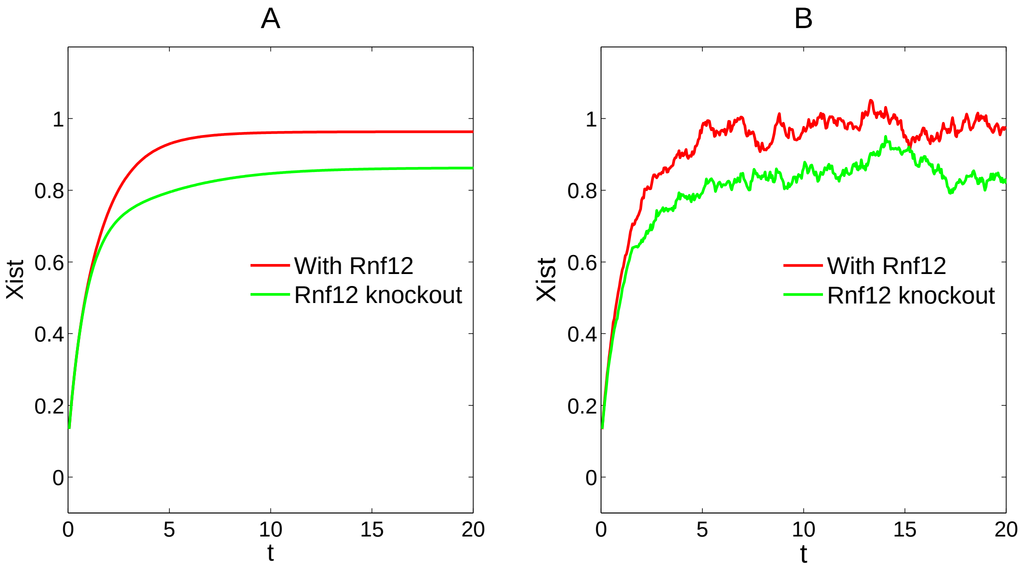

Figure S4. Comparisons of simulation trajectories for Xist activation between the model with RNF12 and without RNF12. (A) Deterministic trajectories. (B) Stochastic trajectories at noise amplitude d=0.05.

Table S1. Parameters of the cross inhibition (CI) model

Symbol	Definition	Value
D1	rate coefficient for the diffusion from Jpx to JpxB	1
D2	rate coefficient for the diffusion from JpxB to Jpx	1
n	Hill coefficient	4
S	threshold for Hill function	0.5
b	represson constant of CTCF to xist	0.84
b2	represson constant between two xist	0.06
k	degradation constant	1
a_{ctcf}	basal synthesis rate for RNA ctcf	1
a_{CTCF}	basal synthesis rate for protein CTCF	1
a_{xist}	basal synthesis rate for RNA xist	0.1
h1	binding constant for jpx and CTCF	100
f1	dissociate constant for jpx and CTCF	1
h2	binding constant for jpx and JC1	100
f2	dissociate constant for jpx and JC1	1
k_{jpx}	degradation constant of jpx	40
k_{xist}	degradation constant of xist	1

Table S2. Parameters of the self-catalyzed binding (SCB) model

Symbol	Definition	Value
n	Hill coefficient	4
S	threshold for Hill function	0.5
a1	self-activation constant of xist	0.5
b	represson constant of CTCF to xist	0.4
k	degradation constant	1
a_{ctcf}	basal synthesis rate for RNA ctcf	1
a_{CTCF}	basal synthesis rate for protein CTCF	1
a_{xist}	basal synthesis rate for RNA xist	0.1
$h1_0$	basal binding constant for jpx and CTCF	0.01
f1	dissociate constant for jpx and CTCF	1
h2	binding constant for jpx and JC1	100
f2	dissociate constant for jpx and JC1	1
k_{jpx}	degradation constant of jpx	0
k_{xist}	degradation constant of xist	1
kh	strength of self-catalyzed binding positive feedback	25
SS	threshold for Hill function of self-activated binding	1
n1	Hill coefficient for self-activated binding of jpx and CTCF	2
Dh_A	Diffusion constant from Jfree to Jpx	0.1
Dh_B	Diffusion constant from Jfree to JpxB	0.1
Df_A	Diffusion constant from Jpx to Jfree	0.01
Df_B	Diffusion constant from JpxB to Jfree	0.01

Table S3. Parameters of the self-enhanced transport (SET) model

Symbol	Definition	Value
n	Hill coefficient	4
S	threshold for Hill function	0.5
a1	self-activation constant of xist	0.5
b	represson constant of CTCF to xist	0.4
k	degradation constant	1
a_{ctcf}	basal synthesis rate for RNA ctcf	1
a_{CTCF}	basal synthesis rate for protein CTCF	1
a_{xist}	basal synthesis rate for RNA xist	0.1
h1	binding constant for jpx and CTCF	100
f1	dissociate constant for jpx and CTCF	1
h2	binding constant for jpx and JC1	100
f2	dissociate constant for jpx and JC1	1
k_{jpx}	degradation constant of jpx	0
k_{xist}	degradation constant of xist	1
kh	strength of self-enhanced transport positive feedback	3
SS	threshold for Hill function of self-activated diffusion	1
n1	Hill coefficient for self-activated diffusion of jpx	2
Dh_0	basal diffusion constant from Jfree to Jpx or JpxB	0.0001
Df_A	Diffusion constant from Jpx to Jfree	0.2
Df_B	Diffusion constant from JpxB to Jfree	0.2

Table S4. Parameters of the expanded SET model (ESET) including Rnf12, Rex1 and Tsix involved regulations, shown in Fig. S3A.

Symbol	Definition	Value
n	Hill coefficient	4
S	threshold for Hill function	0.5
a1	self-activation constant of xist	0.2
b0	repression constant of CTCF to xist	0.8
b	repression constant	0.2
a	activation constant	0.5
k	degradation constant	1
a_{ctcf}	basal synthesis rate for RNA ctcf	1
a_{CTCF}	basal synthesis rate for protein CTCF	1
a_{xist}	basal synthesis rate for RNA xist	0.1
a_{Tsix}	basal synthesis rate for RNA Tsix	0
a_{Rex1}	basal synthesis rate for Protein Rex1	0
a_{Rnf12}	basal synthesis rate for Protein Rnf12	0
h1	binding constant for jpx and CTCF	100
f1	dissociate constant for jpx and CTCF	1
h2	binding constant for jpx and JC1	100
f2	dissociate constant for jpx and JC1	1
k_{jpx}	degradation constant of jpx	0
k_{xist}	degradation constant of xist	1
k_{Tsix}	degradation constant of Tsix	1
k_{Rex1}	degradation constant of Rex1	1
k_{Rnf12}	degradation constant of Rnf12	1
kh	strength of self-enhanced transport positive feedback	3
SS	threshold for Hill function of self-activated diffusion	1
n1	Hill coefficient for self-activated diffusion of jpx	2
Dh_0	basal diffusion constant from Jfree to Jpx or JpxB	0.0001
Df_A	Diffusion constant from Jpx to Jfree	0.2
Df_B	Diffusion constant from JpxB to Jfree	0.2

ODEs for cross inhibition (CI) model

In the following ODEs, Jpx, ctcf, CTCF, xist, JC1, and JC2 represents the corresponding proteins or RNAs in X chromosome 1, and JpxB, ctcfB, CTCFB, xistB, JC1B, and JC2B represents corresponding proteins or RNAs in X chromosome 2. Jfree represent the Free Jpx in the Fig. 1 of main text.

$$djpx/dt = J0 - k_{jpx} * jpx - h1 * jpx * CTCF + (f1 + k_{JC1}) * JC1 \\ - D1 * jpx + D2 * jpxB \tag{1}$$

$$dctcf/dt = a_{ctcf} - k * ctcf \tag{2}$$

$$dCTCF/dt = - k * CTCF + a_{CTCF} * ctcf - h1 * jpx * CTCF + f1 * JC1 - h2 * JC1 * CTCF \\ + f2 * JC2 \tag{3}$$

$$dxist/dt = a_{xist} - k_{xist} * xist + (b * S^n)/(S^n + CTCF^n) \\ * (xist^{n1})/(S^n + xist^n) + (b2 * S^n)/(S^n + xistB^n) \tag{4}$$

$$dJC1/dt = h1 * jpx * CTCF - f1 * JC1 - h2 * JC1 * CTCF + f2 * JC2 - k_{JC1} * JC1 \tag{5}$$

$$dJC2/dt = h2 * JC1 * CTCF - f2 * JC2 - k_{JC2} * JC2 \tag{6}$$

$$djpxB/dt = J0 - k_{jpx} * jpxB - h1 * jpxB * CTCFB + (f1 + k_{JC1B}) * JC1B \\ + D1 * jpx - D2 * jpxB \tag{7}$$

$$dctcfB/dt = a_{ctcf} - k * ctcfB \tag{8}$$

$$dCTCFB/dt = - k * CTCFB + a_{CTCF} * ctcfB - h1 * jpxB * CTCFB + f1 * JC1B - h2 * JC1B * CTCFB \\ + f2 * JC2B \tag{9}$$

$$dxistB/dt = a_{xist} - k_{xist} * xistB + (b * S^n)/(S^n + CTCFB^n) \\ * (xistB^{n1})/(S^{n1} + xistB^{n1}) + (b2 * S^n)/(S^n + xist^n) \tag{10}$$

$$dJC1B/dt = h1 * jpxB * CTCFB - f1 * JC1B - h2 * JC1B * CTCFB + f2 * JC2B - k_{JC1} * JC1B \tag{11}$$

$$dJC2B/dt = h2 * JC1B * CTCFB - f2 * JC2B - k_{JC2} * JC2B \tag{12}$$

ODEs for self-catalyzed binding (SCB) model

$$\begin{aligned} djpx/dt =& J0A - k_{jpx} * jpx - h1 * jpx * CTCF + (f1 + k_{JC1}) * JC1 + k_{JC2} * JC2 \\ & - Df_A * jpx + Dh_A * Jfree \end{aligned} \tag{13}$$

$$dctcf/dt = a_{ctcf} - k * ctcf \tag{14}$$

$$\begin{aligned} dCTCF/dt =& - k * CTCF + a_{CTCF} * ctcf - h1 * jpx * CTCF + f1 * JC1 - h2 * JC1 * CTCF \\ & + f2 * JC2 \end{aligned} \tag{15}$$

$$\begin{aligned} dxist/dt =& a_{xist} - k_{xist} * xist + (b * S^n)/(S^n + CTCF^n) \\ & + a1 * (xist^n)/(S^n + xist^n) \end{aligned} \tag{16}$$

$$dJC1/dt = h1 * jpx * CTCF - f1 * JC1 - h2 * JC1 * CTCF + f2 * JC2 - k_{JC1} * JC1 \tag{17}$$

$$dJC2/dt = h2 * JC1 * CTCF - f2 * JC2 - k_{JC2} * JC2 \tag{18}$$

$$\begin{aligned} djpxB/dt =& J0B - k_{jpx} * jpxB - h1B * jpxB * CTCFB + (f1 + k_{JC1}) * JC1 + k_{JC2} * JC2B \\ & - Df_B * jpxB + Dh_B * Jfree \end{aligned} \tag{19}$$

$$dctcfB/dt = a_{ctcf} - k * ctcfB \tag{20}$$

$$\begin{aligned} dCTCFB/dt =& - k * CTCFB + a_{CTCF} * ctcfB - h1B * jpxB * CTCFB + f1 * JC1B - h2 * JC1B * CTCFB \\ & + f2 * JC2B \end{aligned} \tag{21}$$

$$\begin{aligned} dxistB/dt =& a_{xist} - k_{xist} * xistB + (b * S^n)/(S^n + CTCFB^n) \\ & + a1 * (xistB^n)/(S^n + xistB^n) \end{aligned} \tag{22}$$

$$dJC1B/dt = h1B * jpxB * CTCFB - f1 * JC1B - h2 * JC1B * CTCFB + f2 * JC2B - k_{JC1} * JC1B \tag{23}$$

$$dJC2B/dt = h2 * JC1B * CTCFB - f2 * JC2B - k_{JC2} * JC2B \tag{24}$$

$$dJfree/dt = Df_A * jpx - Dh_A * Jfree + Df_B * jpxB - Dh_B * Jfree \tag{25}$$

$$\begin{aligned} h1 =& h1_0 + kh * JC2^{n1}/(SS^{n1} + JC2^{n1}) \\ h1B =& h1_0 + kh * JC2B^{n1}/(SS^{n1} + JC2B^{n1}) \end{aligned} \tag{26}$$

ODEs for self-enhanced transport (SET) model

$$djpx/dt = J0A - k_{jpx} * jpx - h1 * jpx * CTCF + (f1 + k_{JC1}) * JC1 + k_{JC2} * JC2 - Df_A * jpx + Dh_A * Jfree \quad (27)$$

$$dctcf/dt = a_{ctcf} - k * ctcf \quad (28)$$

$$dCTCF/dt = -k * CTCF + a_{CTCF} * ctcf - h1 * jpx * CTCF + f1 * JC1 - h2 * JC1 * CTCF + f2 * JC2 \quad (29)$$

$$dxist/dt = a_{xist} - k_{xist} * xist + (b * S^n)/(S^n + CTCF^n) + a1 * (xist^n)/(S^{nn} + xist^n) \quad (30)$$

$$dJC1/dt = h1 * jpx * CTCF - f1 * JC1 - h2 * JC1 * CTCF + f2 * JC2 - k_{JC1} * JC1 \quad (31)$$

$$dJC2/dt = h2 * JC1 * CTCF - f2 * JC2 - k_{JC2} * JC2 \quad (32)$$

$$djpxB/dt = J0B - k_{jpx} * jpxB - h1 * jpxB * CTCFB + (f1 + k_{JC1}) * JC1 + k_{JC2} * JC2B - Df_B * jpxB + Dh_B * Jfree \quad (33)$$

$$dctcfB/dt = a_{ctcf} - k * ctcfB \quad (34)$$

$$dCTCFB/dt = -k * CTCFB + a_{CTCF} * ctcfB - h1 * jpxB * CTCFB + f1 * JC1B - h2 * JC1B * CTCFB + f2 * JC2B \quad (35)$$

$$dxistB/dt = a_{xist} - k_{xist} * xistB + (b * S^n)/(S^n + CTCFB^n) + a1 * (xistB^n)/(S^n + xistB^n) \quad (36)$$

$$dJC1B/dt = h1 * jpxB * CTCFB - f1 * JC1B - h2 * JC1B * CTCFB + f2 * JC2B - k_{JC1} * JC1B \quad (37)$$

$$dJC2B/dt = h2 * JC1B * CTCFB - f2 * JC2B - k_{JC2} * JC2B \quad (38)$$

$$dJfree/dt = Df_A * jpx - Dh_A * Jfree + Df_B * jpxB - Dh_B * Jfree \quad (39)$$

$$Dh_A = Dh_0 + kh * JC2^{n1}/(SS^{n1} + JC2^{n1})$$
$$Dh_B = Dh_0 + kh * JC2B^{n1}/(SS^{n1} + JC2B^{n1}) \quad (40)$$

ODEs for the expanded SET model (ESET) shown in Fig. 7 in main text

$$djpx/dt = J0A - k_{jpx} * jpx - h1 * jpx * CTCF + (f1 + k_{JC1}) * JC1 + k_{JC2} * JC2 \\ - Df_A * jpx + Dh_A * Jfree \tag{41}$$

$$dctcf/dt = a_{ctcf} - k * ctcf \tag{42}$$

$$dCTCF/dt = -k * CTCF + a_{CTCF} * ctcf - h1 * jpx * CTCF + f1 * JC1 - h2 * JC1 * CTCF \\ + f2 * JC2 \tag{43}$$

$$dxist/dt = a_{xist} - k_{xist} * xist + (b * S^n)/(S^n + CTCF^n) \\ + a1 * (xist^n)/(S^n + xist^n) \tag{44}$$

$$dJC1/dt = h1 * jpx * CTCF - f1 * JC1 - h2 * JC1 * CTCF + f2 * JC2 - k_{JC1} * JC1 \tag{45}$$

$$dJC2/dt = h2 * JC1 * CTCF - f2 * JC2 - k_{JC2} * JC2 \tag{46}$$

$$dTsix/dt = a_{Tsix} - k_{Tsix} * Tsix + a * (Rex1^n)/(S^n + Rex1^n) \tag{47}$$

$$dRex1/dt = a_{Rex1} - k_{Rex1} * Rex1 + a1 * (Rex1^n)/(S^n + Rex1^n) \\ + (b * S^n)/(S^n + xist^n) + (b * S^n)/(S^n + RNF12^n) \tag{48}$$

$$dRnf12/dt = a_{Rnf12} - k_{Rnf12} * Rnf12 + (b * S^n)/(S^n + xist^n) \\ + (b * S^n)/(S^n + Rex1^n) \tag{49}$$

$$djpxB/dt = J0B - k_{jpx} * jpxB - h1 * jpxB * CTCFB + (f1 + k_{JC1}) * JC1 + k_{JC2} * JC2B \\ - Df_B * jpxB + Dh_B * Jfree \tag{50}$$

$$dctcfB/dt = a_{ctcf} - k * ctcfB \tag{51}$$

$$dCTCFB/dt = -k * CTCFB + a_{CTCF} * ctcfB - h1 * jpxB * CTCFB + f1 * JC1B - h2 * JC1B * CTCFB \\ + f2 * JC2B \quad (52)$$

$$dxistB/dt = a_{xist} - k_{xist} * xistB + (b * S^n)/(S^n + CTCFB^n) \\ + a1 * (xistB^n)/(S^n + xistB^n) \quad (53)$$

$$dJC1B/dt = h1 * jpxB * CTCFB - f1 * JC1B - h2 * JC1B * CTCFB + f2 * JC2B - k_{JC1} * JC1B \quad (54)$$

$$dJC2B/dt = h2 * JC1B * CTCFB - f2 * JC2B - k_{JC2} * JC2B \quad (55)$$

$$dTsixB/dt = a_{Tsix} - k_{Tsix} * TsixB + a * (Rex1B^n)/(S^n + Rex1B^n) \quad (56)$$

$$dRex1B/dt = a_{Rex1} - k_{Rex1} * Rex1B + a1 * (Rex1B^n)/(S^n + Rex1B^n) \\ + (b * S^n)/(S^n + xistB^n) + (b * S^n)/(S^n + RNF12B^n) \quad (57)$$

$$dRnf12B/dt = a_{Rnf12} - k_{Rnf12} * Rnf12B + (b * S^n)/(S^n + xistB^n) \\ + (b * S^n)/(S^n + Rex1B^n) \quad (58)$$

$$dJfree/dt = Df_A * jpx - Dh_A * Jfree + Df_B * jpxB - Dh_B * Jfree \quad (59)$$

$$Dh_A = Dh_0 + kh * JC2^{n1}/(SS^{n1} + JC2^{n1}) \\ Dh_B = Dh_0 + kh * JC2B^{n1}/(SS^{n1} + JC2B^{n1}) \quad (60)$$

References

1. Li C, Wang J (2013) Quantifying cell fate decisions for differentiation and reprogramming of a human stem cell network: Landscape and biological paths. PLoS Comput Biol 9: e1003165.

2. Li C, Wang J (2014) Quantifying the underlying landscape and paths of cancer. J R Soc Interface 10: 20140774.

3. Huang S, Guo Y, May G, Enver T (2007) Bifurcation dynamics of cell fate decision lineage-commitment in bipotent progenitor cells. Dev Biol 305: 695-713.

4. Hellman LM, Fried MG (2007) Electrophoretic mobility shift assay (emsa) for detecting protein–nucleic acid interactions. Nature protocols 2: 1849–1861.

5. Sun S, Del Rosario BC, Szanto A, Ogawa Y, Jeon Y, et al. (2013) Jpx rna activates xist by evicting ctcf. Cell 153: 1537–1551.

6. Gadagkar SR, Call GB (2015) Computational tools for fitting the hill equation to dose–response curves. Journal of pharmacological and toxicological methods 71: 68–76.
